# The moderating effect of altruism on the relationship between occupational stress and turnover intentions: a cross-sectional study of community rehabilitation workers in China

**DOI:** 10.1186/s40359-024-01926-z

**Published:** 2024-08-14

**Authors:** Nian Liu, Yiyang Shu, Wei Lu, Yongshi Lin

**Affiliations:** 1 Department of Sociology, Guangzhou University, Guangzhou, 510006 China; 2 Department of Sociology, The Chinese University of Hong Kong, Hong Kong, 999077 Hong Kong; 3 School of Sociology & Anthropology, Xiamen University, Xiamen, 361005 China; 4 School of Political Science and Law, Wuyi University, Jiangmen, 529020 China; 5 Center for Chinese Studies, The University of California at Berkeley, Berkeley, 94704 USA

**Keywords:** Community rehabilitation worker, Altruism, Occupational stress, Turnover intention, Partial least squares, Moderated mediation

## Abstract

**Background:**

In China, community rehabilitation workers are facing a growing challenge related to heavy occupational stress, which is having an impact on employment turnover. Previous studies have explored the effect of the public service motivation of workers in “helping” jobs on occupational stress or turnover intention, but there is a lack of clarification of the impact of altruism on turnover intention in the case of complex pathways involving various factors.

**Methods:**

A stratified sampling method was used, and a total of 82 community rehabilitation workers who assist disabled people from 34 community health centres in Jiangmen city were included in the study from August to October 2022. The turnover intention, occupational stress, burnout, quality of life, altruism, and certain sociodemographic information of community rehabilitation workers were measured using a structured questionnaire. The partial least squares method was employed to construct and test the structural equation model.

**Results:**

Although altruism had no direct impact on occupational stress or turnover intention, altruism moderated the effect of occupational stress on burnout (*β*_*Mod*_ = −0.208) and quality of life (*β*_*Mod*_ = 0.230) and weakened the mediation of burnout and quality of life between occupational stress and turnover intention.

**Conclusions:**

This study proposes to address the dilemma of “strong function” and “weak specialty” in community rehabilitation services and to conduct positive psychological interventions for community rehabilitation workers through the guidance of altruistic values.

**Supplementary Information:**

The online version contains supplementary material available at 10.1186/s40359-024-01926-z.

## Introduction

According to the China Disabled Persons’ Federation (CDPE), there were 85 million disabled people in China by the end of 2020, accounting for approximately 6.21% of the total population, among whom nearly 50 million were in need of rehabilitation [1]. Most disabled people live in the community and receive community rehabilitation treatment, except a very small number of disabled people receiving hospital rehabilitation treatment in medical institutions [2]. By the end of 2020, there were a total of 10,440 rehabilitation institutions for disabled people in China, including 2,550 rehabilitation institutions (which mainly includes specialized social nursing homes and rehabilitation hospitals) in the CDPE system and 7,890 community-based rehabilitation institutions; there were 295,000 people working in rehabilitation institutions, including 213,000 workers who provided direct rehabilitation services [3]. According to national statistics, 213,000 workers serve more than 50 million disabled people, with a single worker serving an average of 250 disabled people.

Historically, to develop welfare support, the Chinese government has established community rehabilitation institutions for disabled people at the neighbourhood and town levels. Chinese community rehabilitation institutions have experienced development and transformation from community work treatment stations to community health centres [4]. Since 2017, Guangdong Province, as a national pilot area, has rapidly built community health centres throughout the province. In contrast to traditional treatment stations that emphasize vocational rehabilitation, community health centres focus on the rehabilitation of vocational skills for disabled people, training disabled people to live at home, providing daily life care and enhancing their ability to integrate into the community. With the expansion of community health centres in terms of service function and content, compared to traditional treatment stations, higher functional ability and work requirements are considered in the training of community rehabilitation workers.

In contrast to strengthening the composition of a high-quality work team, the turnover of community rehabilitation workers is becoming increasingly serious. Studies show that there is an insufficient number of existing professionals, and turnover rates in China’s community rehabilitation institutions are as high as 60% [5]. Community rehabilitation workers are faced with the development dilemma of high work pressure and unstable teams, which has severely hindered the improvement of the community welfare of people with disabilities in China [6].

Rehabilitation with disabled people is a specialized occupation, and the decision to work in this field is guided by altruistic values [7]. The altruistic behaviour of rehabilitation workers derives from a place of care and compassion for disabled people rather than for social returns, and whether an employee demonstrates altruistic motivation is often an important consideration in the recruitment process [8]. Altruism is people-oriented and helps to motivate workers to invest fully in their potential, cope with work pressure and adjust career challenges [9]. This attitude serves to improve mental health, promote quality of life, and build good professional self-esteem in rehabilitation workers [10].

Some studies have explored the relationships between altruistic values and employee pressure, quality of life and turnover intention for helping professionals, such as medical and teaching staff [11–13]. However, these studies concentrate on the independent relationship between altruism and other factors, while few studies have comprehensively considered the role of altruism in cases of the complex mechanisms of various factors. This study focuses on the pathways between altruism and work pressure, burnout, quality of life and turnover intention. The aim is to clarify how the altruistic values of community rehabilitation workers affect their turnover intentions, and corresponding research countermeasures are proposed.

## Literature and hypotheses

The concepts of altruism and public service motivation are closely related. The former is more general, with motivation as its core attribute, and can be understood as the broad concept underpinning public service motivation [14]. Public service motivation, as a type of incentive structure of individual resources and organizations, plays a crucial role in explaining work-related attitudes and behaviours, such as work stress, burnout, turnover intention and other occupation-related variables. Self-regulation theory emphasizes how individuals manage and control their behaviour, emotions, and motivations through self-observation, self-judgement, and self-response [15]. Through self-regulation, individuals can exert a certain level of control over their thoughts, emotions, and behaviours to adapt to various environments and challenges. As a projective self-defence mechanism, altruism is an attitude towards life that individuals display by vigorously controlling their own needs or actions to fulfil the desires of others [16]. In the workplace, altruism can be seen as a positive coping strategy that helps employees better manage job stress and reduce their intention to leave. Specifically, the altruistic tendencies of community rehabilitation workers can be viewed as a resource or strategy for self-regulation [17], which assists individuals in more effectively dealing with job stress and lowering their turnover intentions.

Altruism is negatively correlated with turnover intention. Employees with high altruism are less likely to express turnover intention than are those with low altruism [18, 19]. Fostering altruism in an organization can effectively inhibit employees’ turnover intention, thus reducing the loss of employees [12]. Moreover, public service motivation, as an individual motivation to serve others and society, is also regarded as an important resource for individuals to overcome pressure [20]; the altruistic behaviours of helping others can effectively improve work efficiency and quality [21]. Therefore, from the perspective of public service motivation, namely, altruism, this study discusses the impact of altruistic value tendencies on work pressure and turnover intention and proposes two hypotheses:

### H1

*Altruism negatively affects occupational stress. The stronger the tendency towards altruism is*,* the lower the occupational stress.*

### H2

*Altruism negatively affects turnover intention. The stronger the tendency towards altruism is*,* the lower the turnover intention.*

Occupational stress is a syndrome characterized by emotional exhaustion, depersonalization and a decreased sense of personal accomplishment among people in the work environment; this stress can lead to irritability, sleep disorders, hypertension, anxiety and chronic cynicism [22]. To explain the source of pressure according to resource protection theory, when the need for work exceeds individuals’ ability and resources, individuals will be deprived of their own personal resources, leading to a sense of threat and instability, and pressure is generated accordingly, damaging the physical and psychological health of employees [23, 24]. Fukui, Rollins and Salyers [25] suggested that the effect of occupational stress on turnover intention was much greater than those of the organization and working environment. In contrast, when individuals’ sense of pressure is reduced, they will experience happiness and move towards an autonomous motivational state, which will further encourage them to pursue their interests, goals and experiences at work and reduce their turnover intention [26].

Burnout is a progressive psychological response to chronic occupational stress, including emotional exhaustion, depersonalization and a decreased sense of personal accomplishment [27]. Studies show that burnout is directly caused by work stressors [24, 28], and employees experiencing burnout are more likely than others to exhibit withdrawal behaviour (such as absenteeism) or refuse to socialize with other colleagues [29] and are more inclined to quit [28, 30]. Compared with other employees, people in the public service industry are more likely to suffer from burnout [31], and a high level of exhaustion for an extended period is more likely to increase employee turnover intention. When an individual’s work pressure increases, resulting in emotional exhaustion and work-life conflicts, personal health, including physical and psychological health, will also improve, and the personal health of employees is closely related to their quality of working life [32]. Through a survey of medical workers, Mosadeghrad, Ferlie and Rosenberg [33] found that occupational stress poses a serious threat to the quality of working life and eventually leads to employee turnover, which increases the turnover loss of the organization. Xu, Chen and Shao [34] indicated that the quality of working life was negatively correlated with turnover intention. The greater the quality of working life is, the lower the turnover intention. In short, turnover intention is directly or indirectly affected by work pressure, and burnout and quality of life play important mediating roles in the relationship between work pressure and turnover intention. Therefore, this paper proposes the following three hypotheses:

### H3

*Occupational stress positively affects turnover intention. The greater the occupational stress is*,* the stronger the turnover intention.*

### H4

*Occupational stress indirectly affects turnover intention through a positive effect on burnout*,* and burnout plays a mediating role.*

### H5

*Occupational stress indirectly affects turnover intention through a negative effect on quality of life*,* and quality of life plays a mediating role.*

Public service motivation, as an individual motivation to serve others and society, is also regarded as an important resource for individuals to overcome pressure [20]. According to self-regulation theory, individuals set coping strategies based on their own values and goals when faced with stress [35]. Individuals make value judgements about their behaviour, emotions, and motivations based on the results of self-observation, determining whether they align with their own goals and expectations. Subsequently, they take appropriate measures to adjust to the challenges they face to achieve their set objectives [15]. Therefore, community rehabilitation workers with a strong tendency towards altruism are likely to adopt positive self-regulation strategies to cope with job stress. These strategies may include adjusting work attitudes, seeking social support, or employing other methods that help alleviate stress [36]. This positive self-regulation helps to reduce occupational burnout and poor quality of life caused by job stress, which in turn reduces the intention to leave. An altruistic orientation can help workers focus more on the needs and welfare of disabled individuals, thereby reducing their perception of job stress. When workers concentrate on helping others, they are more likely to find meaning and value in their work, which in turn reduces the negative effects of job stress. Furthermore, an altruistic orientation can help community disability support workers maintain a positive attitude and motivation. They are more likely to view their work as a mission and responsibility rather than just a job. This sense of mission can inspire workers’ intrinsic motivation, making them more willing to invest time and effort to address difficulties at work [37]. Therefore, an altruistic orientation can ultimately reduce workers’ turnover intentions.

Individuals with high public service motivation are more inclined to choose industries that can fulfil their public service needs in the long run because their values are consistent with the missions of public service organizations. Even under heavy pressure, they are also more willing to develop professionally in the public service industry over a long period and are less likely to quit [32, 38]. Other studies have shown that [13, 39], under high pressure, individuals with high public service motivation have greater psychological health than those with low public service motivation and are more likely to have a higher quality of working life, which may lead to an autonomous motivational orientation. At the same time, individuals with high public service motivation will regard stressors as a challenge and an opportunity to do good for others and society; these individuals will then focus on their internal desires to better manage the emotions related to stressors, choose appropriate solutions and reduce the potential negative emotional impact of stress, thus reducing their sense of burnout [22]. Therefore, this paper hypothesizes that altruistic service motivation also moderates the direct effect of work pressure on turnover intention and on the effect of work pressure on turnover intention through burnout and quality of life:

### H6

*: Altruism moderates the direct effect of occupational stress on turnover intention*,* and altruism weakens this effect.*

### H7

*: Altruism has a moderating effect on the relationship between occupational stress and burnout*,* and altruism weakens the effect of occupational stress on burnout*,* which in turn affects the mediating effect of burnout on the relationship between occupational stress and turnover intention.*

### H8

*Altruism has a moderating effect on the relationship between occupational stress and quality of life*,* and altruism weakens the effect of occupational stress on quality of life*,* which in turn affects the mediating effect of quality of life between occupational stress and turnover intention.*

**Fig. 1:**
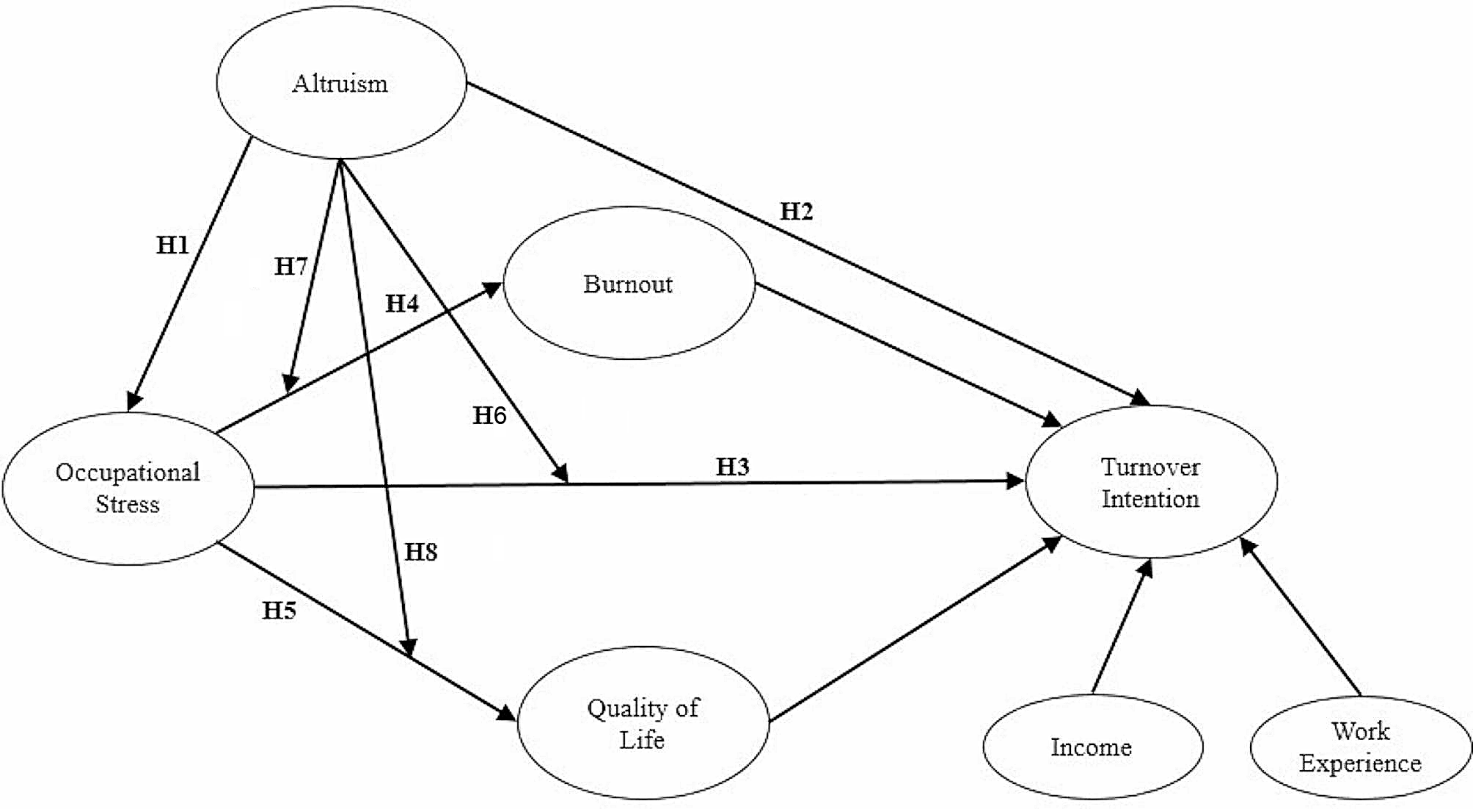
Framework of hypothesis

Based on the above research hypotheses, this study takes turnover intention as the dependent variable to construct a structural path model of altruism, work pressure, burnout and quality of life that can affect the turnover intention of community rehabilitation workers (see Fig. 1). In addition, employees’ salary and working years affect their turnover intention, and a higher salary and longer working years reduce their turnover intention [40]. Clearly, employees’ salary is negatively correlated with their turnover intention; that is, the lower the employees’ salary is, the greater their turnover intention [11]. Studies have shown that employees’ working years are negatively correlated with their turnover intention; that is, the longer the employees’ working years are, the lower their turnover intention is [41]. This study introduces the salary and working years of community rehabilitation workers into the structural model as control variables to eliminate their effects on turnover intention.

## Methods

### Sampling and sample size

This study included all community rehabilitation workers in Jiangmen as the sample population. Jiangmen city, in central Guangdong Province, is a relatively developed area of economy and community service in China. This study began with a sampling survey from August to October 2022, and the survey process was divided into two steps: community health centre sampling and community rehabilitation worker sampling.

The first step was to select the community health centre. In 2018, Jiangmen began a vigorous effort to build community health centres at the neighbourhood and town levels, which are mainly responsible for providing vocational rehabilitation, rehabilitation function training, home living ability training and other daytime care services (without accommodation) for people with mental, intellectual and physical disabilities. By the end of 2021, a total of 37 community health centres had been built in Jiangmen city. Among them, 3 health centres specialize in providing rehabilitation services for people with vision, hearing and speech disabilities and are directly affiliated with the Jiangmen Disabled Persons’ Federation [42] and not under the jurisdiction of streets and towns. These centres were not included in this survey due to their special ownership. The remaining 34 community health centres were included.

The second step was to select community rehabilitation workers. In this study, community rehabilitation workers refer to (1) full-time workers who work in community health centres and sign formal labour contracts with the centres, excluding part-time workers; (2) professionals who are directly engaged in rehabilitation services for disabled people, including those who provide professional services such as life guidance, vocational rehabilitation and psychological counselling in the posts of social work, rehabilitation therapists and physiotherapists, excluding administrative, logistic and managerial personnel. Generally, one community health centre employs approximately 4–10 rehabilitation workers. Considering the limited research funds, this study selected 5 as a limit, and if the number of workers was ≤ 5, 2 people were selected; if the number of workers was < 10, 3 people were selected; and if the number of workers was ≥ 10, 4 people were selected. A corresponding number of rehabilitation workers were randomly selected based on the order of their work card number, with an interval of “3” (if refused, the subsequent worker was selected), and a structured questionnaire survey was conducted.

This study was approved by the Ethics Committee of the School of Public Administration of Guangzhou University, and the relevant survey procedures were in line with the requirements of the Declaration of Helsinki. The questionnaire survey was conducted in a separate room, and the respondents could choose to complete the questionnaire through question-and-answer or self-reports. After the questionnaire was completed, the researcher immediately recovered and sealed it for unified data entry. The data from this research do not reveal any personal identifying information related to the respondents.

In this survey, a total of 91 community rehabilitation workers were selected from 34 community health centres in Jiangmen city. Among them, 6 people were removed due to urgent work tasks during the questionnaire interview, and 3 questionnaires were not filled out. Finally, 82 valid questionnaires were recovered, for a valid rate of 90.1% (82/91). During the same period, there were 423 community rehabilitation workers in the community health centres in Jiangmen city [42], and the proportion of research samples accounted for 19.4% of the total number of community rehabilitation workers (82/423). Among the 82 community rehabilitation workers selected, 71 were female and 11 were male; their ages ranged from 22 to 59, with an average age of 34.5; 72 people had local registered residences, and 10 people had registered residences in the province.

The small sample size is one of the major limitations of this study. First, this is the first survey on the mental health status of community rehabilitation workers in China, and this group is widely distributed across communities with heavy daily workloads. Due to time and financial constraints, it was difficult to further expand the sample size. Second, the sample is representative in terms of gender, age, and household registration, which can reflect the characteristics of the target group (Jiangmen Disabled Persons’ Federation, 2022). In addition, we conducted a statistical power analysis, and the results showed that our sample size was sufficient to detect the expected effect size (see Table 1). In conclusion, a sample size of 82 community rehabilitation workers was sufficient to achieve our research objectives.

### Variables

#### Turnover intention (dependent variable)

This variable mainly measures the intention of community rehabilitation workers to leave the community health centre. We refer to the measurement approach used by Abrams, Ando and Hinkle [43] for the turnover intention of enterprise employees, including 4 items scored from 1 to 5 (1 = strongly disagree, 5 = strongly agree), which are as follows: having the idea to leave, expecting to leave within the next few years, considering leaving, and leaving only upon retirement (reverse item). The 4 items together constitute the latent variable of turnover intention, and the load of the standardized factor of items ranges from 0.710 to 0.898 (see Table 2) with the internal consistency reliability of the scale reaching Cronbach’s α = 0.825, the constituent reliability CR = 0.889 and the convergent validity AVE = 0.668. According to the suggestions of Fornell and Larcker [44], the turnover intention factor has good reliability and convergent validity.

**Table 1:** Moderating effects of Altruism

Altruism as Moderator	Estimate	SD	T Value	*P* Value	Bootstrapping	
					Bias Corrected	
					2.5%	97.5%
**Moderating Effect**						
Occupational Stress -> Turnover Intention	− 0.099	0.087	1.145	0.252	− 0.272	0.069
Occupational Stress -> Burnout	− 0.208	0.054	3.826	< 0.001	− 0.303	− 0.091
Occupational Stress -> Quality of Life	0.230	0.078	2.965	0.003	0.068	0.371
**Moderated Mediation**						
Occupational Stress -> Burnout-> Turnover Intention	− 0.054	0.037	1.472	0.141	− 0.149	− 0.001
Occupational Stress -> Quality of Life -> Turnover Intention	− 0.047	0.033	1.407	0.160	− 0.129	− 0.001

**Table 2:** Factor characteristics of the measurement model: reliability and convergence

Factors	Items	Factor Loadings	*P* Value	Cronbach’s α	Composite Reliability (CR)	Average Variance Extracted (AVE)
Turnover Intention	4	0.710-0.898	< 0.001	0.825	0.889	0.668
Occupational Stress	5	0.760-0.931	< 0.001	0.910	0.935	0.742
Burnout	3	0.636-0.857	< 0.001	0.506	0.814	0.597
Quality of Life	4	0.708-0.850	< 0.001	0.788	0.871	0.630
Altruism	4	0.688-0.787	< 0.001	0.725	0.827	0.545
Work Experience	1	1.000	-	-	-	-
Income	1	1.000	-	-	-	-

“-” means that work experience and income are single indicator factors and are not applicable to this statistical index

#### Occupational stress (independent variable)

The occupational stress scale developed by House, Wells, Landerman, et al. [45] was adopted and translated into Chinese by Chen Wei-Qing, Wong and Yu [46] to measure the occupational stress of industrial workers. The scale consists of 15 items scored from 1 to 5 (1 = never, 5 = always). The higher the score is, the greater the work pressure. The scale involves 5 dimensions—responsibility pressure, quality concern, role conflict, job vs. nonjob conflict and workload—each of which includes 3 items. Since the sample size of this study is small, the item parcelling method was adopted to make the parameter estimation relatively stable [47, 48]. The 3 items in each dimension were summed and averaged, and the average score was used to represent this dimension. Finally, after item parcelling, 5 indicators representing 5 dimensions of occupational stress were used to construct latent variables of occupational stress. The standardized factor loadings range from 0.760 to 0.931, with Cronbach’s α = 0.910, CR = 0.935, and AVE = 0.742, and the occupational stress factor has good reliability and convergent validity.

#### Burnout and quality of life (mediating variables)

Burnout refers to a phenomenon of psychological exhaustion caused by work [49]. This study used the Maslach Burnout Inventory General Survey (MBI-GS) [50] to measure burnout. The MBI-GS has a total of 16 items scored from 0 to 6, which are divided into three dimensions: 5 questions for exhaustion, 5 questions for cynicism, and 6 questions for low professional efficiency. The higher the score is, the greater the burnout. Item parcelling was used to package the items of each dimension, and 3 indicators representing 3 dimensions were used to construct a latent variable of burnout. The standardized factor loadings ranged from 0.636 to 0.857, with Cronbach’s α = 506, CR = 0.814 and AVE = 0.597. Quality of life was measured using the Quality of Life Assessment Scale (QOLAS) [51]. The QOLAS consists of 16 items scored from 1 to 5, covering four dimensions: physical, psychological, social and functional. Item parcelling was also used, and the standardized factor loadings of the 4 dimensions ranged from 0.708 to 0.850, with Cronbach’s α = 788, CR = 0.871 and AVE = 0.630. Both burnout and quality of life factors have good reliability and convergent validity.

#### Altruism (independent and moderating variables)

Altruism refers to the degree to which individuals are sincerely sympathetic and caring of others [52]. The measurement of philosophies of human nature can be used to measure altruism [53]. This study uses 4 items ranging from 1 to 5 (1 = strongly disagree, 5 = strongly agree) to construct latent variables of altruism. The 4 items are as follows: most people would help those in distress, most people would sympathize with and assist those in distress, most people would sincerely care about the difficulties of others, and most people would help people in trouble on the roadside. The standardized factor loadings of the items ranged from 0.688 to 0.787, with Cronbach’s α of 0.725, a CR of 0.827 and an AVE of 0.545.

#### Work experience and monthly income (control variables)

Work experience refers to the duration (in “months”) that the respondents have engaged in rehabilitation services in community health centres. Since the duration shows an abnormal distribution, it was converted into a natural logarithm and then included in the structural model [54] to reduce the bias of the inferred results. Monthly income refers to the average after-tax monthly income (RMB) of the respondents in the past year, and the original value is directly entered into the model.

The discriminative validity of each factor was investigated, as shown in Table 3. According to the Fornell & Larcker criterion [55], the AVE square root value of each factor was compared to the correlation coefficients of other factors, and the former was greater than the latter; at the same time, the HTMT coefficient between the two factors was less than 0.85, indicating good discriminative validity between all factors [56]. In conclusion, all the factors in this paper have good reliability and validity.

**Table 3:** Factor characteristics of the Measurement Model: discriminative validity

Factors	Discriminative Validity													
	Turnover Intention		Occupational Stress		Burnout		Quality of Life		Altruism		Work Experience		Income	
Turnover Intention	.***818***													
Occupational Stress	0.477	(0.544)	***0.861***											
Burnout	0.564	(0.762)	0.593	(0.753)	***0.773***									
Quality of Life	− 0.465	(0.554)	− 0.321	(0.360)	− 0.568	(0.784)	***0.793***							
Altruism	− 0.274	(0.325)	− 0.250	(0.292)	− 0.465	(0.664)	0.470	(0.614)	***0.738***					
Work Experience	− 0.298	(0.335)	− 0.115	(0.127)	− 0.121	(0.153)	0.083	(0.106)	− 0.041	(0.177)	-			
Income	− 0.171	(0.181)	− 0.016	(0.076)	0.013	(0.073)	− 0.063	(0.094)	− 0.042	(0.081)	− 0.049	(0.049)	-	

The figures (bold and italic font) on the diagonal are the AVE square root values; the figures in the inferior triangle are the Pearson correlation coefficients; and the values in the “()” are the HTMT values. “-” means that work experience and income are single indicator factors without AVE values

Since all data in this study come from respondents’ answers to the same questionnaire, there may be common method variance (CMV), which will lead to deviation from the real relationship between variables [57]. First, in the design of the questionnaire, the item meaning concealment method [58] was used to proactively prevent CMV. Second, before the model analysis, the CMV was tested. (1) Harman’s one-factor method was used to test CMV, and a common factor was set to be extracted (without subextraction). The explained variation caused by factor 1 was 32.494%; with 50% as the criterion [59], CMV can be preliminarily judged to be absent. (2) The Unmeasured Latent Method Construct (ULMC) method was used in SmartPLS [60] to calculate the values of the mean of substantial factor explanatory variance (R1^2^) and the common method variance (R2^2^), which were 0.695 and 0.022, respectively, and R1^2^:R2^2^ = 32:1 (see Appendix 1). According to the guidelines presented by Liang, Saraf and Xue [61], CMV should not be a serious concern in this study.

### Statistical methods

This study used SmartPLS 3.3.2 (partial least squares) software for statistical analysis. Based on the variance of the observed variables of the structural equation model, PLS statistical analysis estimates the model parameters using partial least squares in combination with principal component analysis and multiple regression analysis to maximize the predictive ability of the model. In contrast to covariance-based structural equation model analysis [62], PLS has several unique advantages: (1) there is no need for analysis data to conform to a multivariate normal distribution, (2) it is especially suitable for model prediction to maximize the explanatory ability of endogenous variables [63], and (3) PLS is especially suitable for small sample studies given that the parameter estimation is consistent [64]. In recent years, the PLS statistical analysis method and its application software have been used more broadly in scientific research [65]. Combined with the characteristics of PLS statistical analysis, SmartPLS software is used for the following reasons: (1) the structural model constructed in this study is relatively complex, involving mediating effects and moderating effects; (2) the sample size of this study is only 82 people, and the data distribution may not be typical; and (3) the focus of the study is to explore the pathway effects of altruistic values, occupational stress and other factors of community rehabilitation workers on turnover intention. Under the framework of the model path, PLS statistical analysis can maximize the explanatory ability of turnover intention. Therefore, this study used SmartPLS software for the statistical analysis of the model. In addition, to make the research conclusions more scientific, this study abandons the use of the *P* value for binary classification [66] but adopts the multiclassification proposal of Muff, Nilsen, O’Hara, et al. [67] to test the research hypotheses.

## Results

### Working situation of community rehabilitation workers

The indicator scores of each factor were arithmetically summed and averaged to describe the group characteristics of the community rehabilitation workers. As shown in Table 4, the overall turnover intention of this group is not high, with a mean value of M = 2.186 (SD = 0.772), which is at a medium or low level; similarly, this group has moderate occupational stress (M = 2.133) and low burnout (M = 1.373). The overall quality of life of the community rehabilitation workers was good (M = 4.667), while their tendency towards a high level of altruistic value was relatively clear (M = 4.012). In conclusion, community rehabilitation workers have a good mental outlook, good health status and a tendency towards greater altruistic values.

**Table 4:** Working status of community rehabilitation workers

Factors	Mean	SD	Median	Min	Max	Skewness	Kurtosis
Turnover Intention	2.186	0.772	2.250	1.000	5.000	− 0.104	− 0.719
Occupational Stress	2.133	0.672	2.200	1.000	5.000	0.655	1.394
Burnout	1.373	0.966	1.256	0.000	6.000	0.182	-1.211
Quality of Life	4.667	0.450	4.833	1.000	5.000	-1.483	1.257
Altruism	4.012	0.565	4.000	1.000	5.000	0.359	− 0.628
Work Experience^1^	41	52	24	1	300	3.187	12.677
Income^2^	2804	949	2950	1200	6000	0.813	0.954

SD = standard deviation. ^1^ The original data time duration without Ln conversion, and the unit is “month”. ^2^The average monthly income in the past year, in RMB

From the perspective of work experience, the average working experience is 3.5 years (41 months), but there is great variation among individuals, including freshmen who have only been in the centre for 1 month and senior employees who have provided rehabilitation services for 300 months. Overall, community rehabilitation workers have a low salary, with a mean of RMB 2,804 yuan/month. Compared with the average salary of on-post workers in Jiangmen city at the end of 2020, which was RMB 7,352 yuan/month [68], the salary of community rehabilitation workers was much lower than the social average in the same period. It is urgent that the salaries of community rehabilitation workers increase.

### Structural path and mediating effects of burnout and quality of life

The path coefficients among the factors were estimated and tested based on the research framework. Since the PLS software was presented with standardized coefficients, the bootstrapping method was used to repeat sampling 5,000 iterations to obtain the standard deviation, thus calculating its *P* value. As shown in Table 5, there is no evidence that altruism has a direct effect on occupational stress and turnover intention, and its path coefficients (standardized) *β* are − 0.250 (*p* = .114) and 0.001 (*p* = .993), respectively. There is strong evidence that occupational stress has a positive effect on burnout (*β* = 0.593, *p* < .001) and a negative effect on quality of life (*β* = − 0.321, *p* = .001). There is moderate evidence that occupational stress (*β* = 0.203, *p* = .048) and burnout (*β* = 0.289, *p* = .042) have a positive effect on turnover intention, and quality of life has a negative effect on turnover intention (*β* = − 0.229, *p* = .027). The work experience (after Ln conversion) and average monthly income of community rehabilitation workers are used as control variables in the structural model; these variables have moderate negative effects on turnover intention. The path coefficients *β* are − 0.231 (*p* = .011) and − 0.198 (*p* = .020), respectively, indicating that the longer the working duration is, the greater the salary and the lower the turnover intention, which plays a controlling role in the structural model.

**Table 5:** Structural model path coefficients

Path Coefficient	Estimate	SD	T Value	*P* Value	Bootstrapping	
					Bias Corrected	
					2.5%	97.5%
Altruism -> Occupational Stress	− 0.250	0.158	1.579	0.114	− 0.519	0.190
Altruism -> Turnover Intention	0.001	0.115	0.008	0.993	− 0.191	0.277
Occupational Stress -> Turnover Intention	0.203	0.103	1.979	0.048	0.008	0.412
Occupational Stress -> Burnout	0.593	0.067	8.885	< 0.001	0.442	0.710
Burnout -> Turnover Intention	0.289	0.142	2.039	0.042	0.034	0.601
Occupational Stress -> Quality of Life	− 0.321	0.101	3.179	0.001	− 0.502	− 0.109
Quality of Life -> Turnover Intention	− 0.229	0.103	2.216	0.027	− 0.428	− 0.029
Path of Control Variables						
Work Experience -> Turnover Intention	− 0.231	0.091	2.543	0.011	− 0.411	− 0.057
Income -> Turnover Intention	− 0.198	0.085	2.336	0.020	− 0.350	− 0.013

SD = standard deviation

After 5000 bootstrapping repeated samples, the bias-corrected estimate interval under the 95% confidence interval did not contain 0, which indicates that the mediating effects of burnout and quality of life both exist. Burnout plays a partial mediating role in the effect of work pressure on turnover intention (see Table 6), and the estimated value of the mediating effect *IE* is 0.171 (*p* = .046); similarly, in another mediating path, quality of life also plays a partial mediating role (*IE* = 0.074, *p* = .081). Comparatively speaking, the mediating effect of burnout is stronger than that of quality of life, and there is weak evidence that a mediating effect of quality of life exists.

**Table 6:** Mediating effects of Burnout and Quality of Life and Total effects

Indirect Effects	Estimate	SD	T Value	*P* Value	Bootstrapping	
					Bias Corrected	
					2.5%	97.5%
**Burnout (as Mediator)**						
Occupational Stress -> Turnover Intention	0.171	0.086	1.997	0.046	0.022	0.351
**Quality of Life (as Mediator)**						
Occupational Stress -> Turnover Intention	0.074	0.042	1.747	0.081	0.008	0.172
**Direct Effects**						
Occupational Stress -> Turnover Intention	0.203	0.103	1.979	0.048	0.008	0.412
**Total Effects**						
Occupational Stress -> Turnover Intention	0.448	0.085	5.306	< 0.001	0.276	0.605

Finally, the overall validity of the model was investigated: the total effect of occupational stress on turnover intention in the structural model *TE* is 0.448 (*p* < .001); if the effect of control variables is accounted for, the overall explanatory power of the structural model on turnover intention *R*^*2*^ is 0.469 (*p* < .001), and the model has moderate explanatory power and good practical value [69]. The blindfolding method was used to calculate turnover intention *Q*^*2*^ *=* 0.283 > 0.000, indicating that the model has good predictive relevance for the turnover intention of community rehabilitation workers. The SRMR model fit index is 0.080 < 0.100, and the structural model is acceptable [70].

### Moderating role of altruism and moderated mediation

Altruism is used as a moderating variable in the structural model. As shown in Table 1, there is no evidence that altruism has a moderating role on the direct effect between occupational stress and turnover intention (*β* = − 0.099), and the 95% bias-corrected estimated interval (-0.272-0.069) contains 0; however, altruism can regulate the regression slopes of occupational stress on burnout and quality of life. Altruism negatively moderates the effect of occupational stress on burnout (*β* = − 0.208), indicating that altruism weakens the effect of occupational stress on burnout. The regression slope of occupational stress on burnout decreases by 0.208 standard deviations for every 1 standard deviation increase in the altruism value of community rehabilitation workers. Under the moderating effect of altruism, the same occupational stress will lead to less burnout, and turnover intention will also decrease. The mediating effect of burnout is reduced, with a moderated mediation effect of − 0.054.

Meanwhile, altruism positively moderates the effect of work pressure on quality of life (*β* = 0.230). Since the effect of occupational stress on quality of life is negative (see Table 4, *β* = − 0.229), this effect indicates that altruism can reduce such a negative effect; that is, the effect of occupational stress on quality of life will increase by 230 standard deviations for every 1 standard deviation increase in altruism. The effect of work pressure on turnover intention through quality of life will also be weakened due to the moderating effect of altruism. The moderated mediation effect of quality of life was − 0.047. Overall, altruism moderates the effect of work pressure on burnout and quality of life and weakens the mediating role of burnout and quality of life between work pressure and turnover intention.

## Discussions

According to the above findings, the team of community rehabilitation workers does not have much work experience, and their salary is far lower than the social average. This group has a strong altruistic tendency with moderate work pressure, good quality of life and low turnover intention.

When work experience and income are controlled for, altruistic values do not directly affect either occupational stress (H1 rejected) or turnover intention (H2 rejected). Occupational stress has a direct positive effect on turnover intention (H3 supported). The greater the occupational stress, the greater the intention to leave the community health centre. Occupational stress has a positive effect on burnout and a negative effect on quality of life and indirectly affects the turnover intention of community rehabilitation workers through the mediating role of burnout (H4 supported) and quality of life (H5 supported). The total indirect effect of burnout and quality of life *TIE* is 0.245 (see Tables 6 and 0.171, 0.074), which is greater than the direct effect of occupational stress on turnover intention (see Table 5, DE, 0.203). The mediating role of burnout is much stronger than that of quality of life (see Table 5, 0.171 > 0.074).

**Fig. 2:**
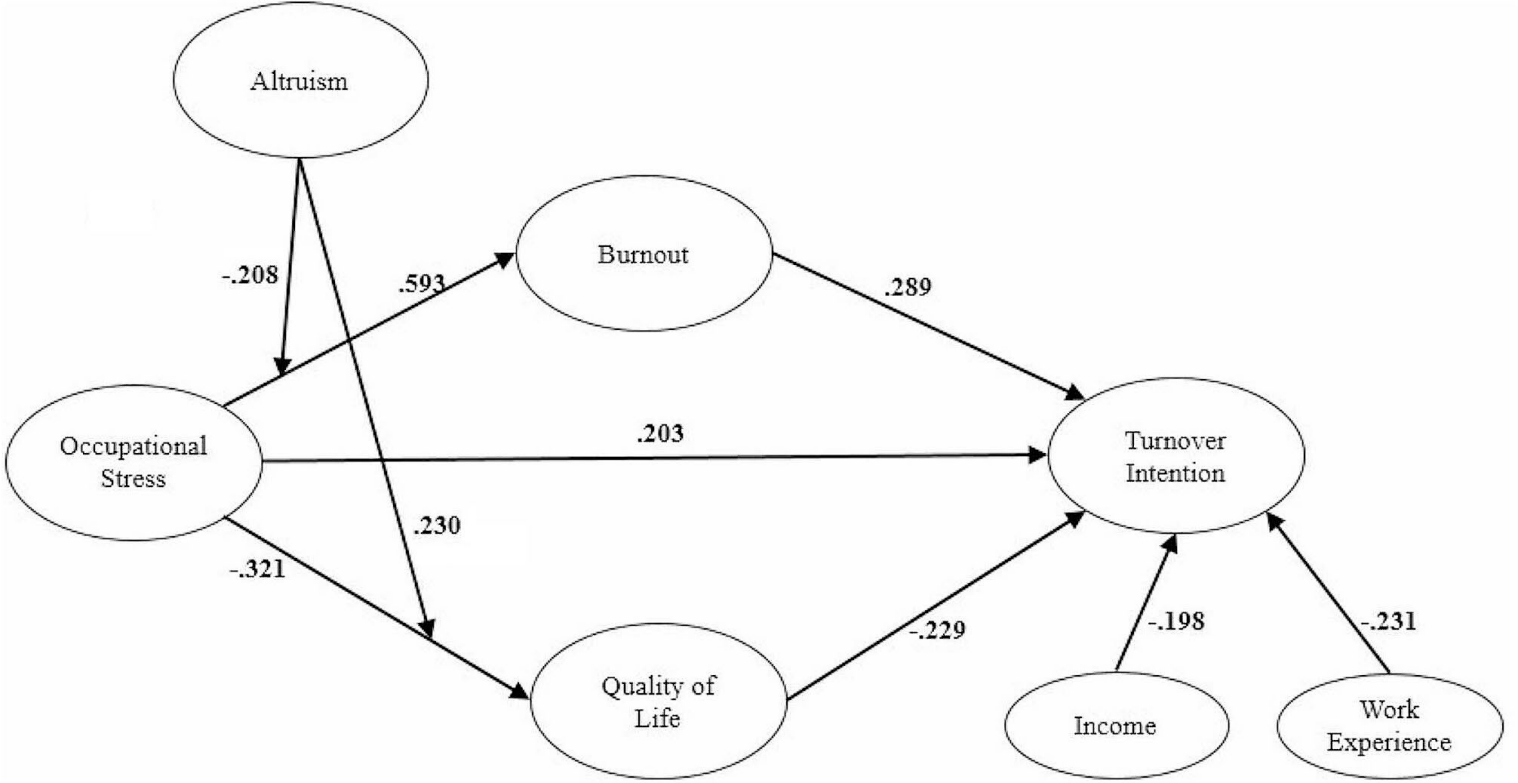
Pathway of conclusions

With altruism as the moderating variable, altruism negatively moderates the positive effect of occupational stress on burnout (see Table 6, *β* = − 0.208), indicating that altruism weakens burnout caused by occupational stress. Altruism positively moderates the negative effect of occupational stress on quality of life (see Table 6, *β* = 0.230), indicating that altruism will counteract the reduction in quality of life caused by occupational stress. By moderating the partial path of the mediating effect of burnout (H7 supported) and quality of life (H8 supported), altruism further moderates the indirect effects of occupational stress on turnover intention. Altruism has no moderating role in the direct effect of occupational stress on turnover intention (H6 rejected). The final structural pathways of the model are shown in Fig. 2.

### Nondirect effect of altruism on work pressure and turnover intention

Bright [9] noted that the public service motivation of public service personnel has no significant effect on job satisfaction and turnover intention when controlling for person-organization fit; similarly, the altruism value of community rehabilitation workers has no direct effect on occupational stress and turnover intention when controlling for work experience and income. As mentioned above, according to national data, there is a serious shortage of people available to serve as Chinese community rehabilitation workers. Workers face great pressure in their daily work to provide rehabilitation services for disabled people. It is necessary to solve the actual conflicts of interest under these pressures, and we cannot rely only on the persistence of the sense of value in altruism because “feelings” will not alleviate the pressures faced by these workers. When community rehabilitation workers feel uncomfortable under occupational stress or have turnover intentions, it is impossible to play a direct and effective role in easing this discomfort by merely emphasizing the dedicated spirit of professionals and highlighting the sense of ethics and value of the position, as this may have a potentially negative effect. Davis, Stazyk, Kochenour, et al. [20] studied the service motivation of public sector personnel by using survey data from the 2010 U.S. Merit Principles, and the results showed that individuals whose public service motivation was higher than average experienced more obvious negative emotions and relative deprivation when they perceived the aggravation of conflicts at the workplace, which increased their willingness to separate from the organization. From a practical point of view, the emphasis on altruism in the profession of helping others needs to be designed through a more scientific path. In this study, altruism exerts a distal effect on turnover intention by moderating the effect of work pressure on burnout and quality of life. The work to help the disabled is first a “job” and then “professional work to help the disabled”. Only when the occupational needs of workers are satisfied and the characteristics of occupational development are affirmed can altruism play a role in stabilizing the team and reducing turnover intention.

### The mediating role of burnout and quality of life on turnover intention behind “strong function” and “weak professional”

In addition to directly affecting turnover intention, the work pressure of community rehabilitation workers indirectly affects their turnover intention via burnout and quality of life. This means that the effect of other psychological and behavioural reactions caused by work pressure on turnover intention cannot be ignored.

The occupational stress of community rehabilitation workers mainly comes from the “strong function” demand of community rehabilitation services that cannot be responded to by workers’ “weak professional” ability. The function of the Chinese community health centre has not only emphasized vocational rehabilitation but also highlighted the daily life functions of disabled people, as well as the simulation training of social functions such as returning to society and integrating into the community. The expansion of multiple service functions and content brings forth greater requirements of “strong function” for community rehabilitation workers as service providers. There are two main sources of community rehabilitation workers: one is the internal posttransfer of staff in grassroots organizations or civil organizations, and the other is social recruitment, both of which pose challenges in setting rigid professional background requirements. As a result, recruiters only conduct an assessment of basic vocational skills [71], thus reflecting the phenomenon of “weak professionalism” for community rehabilitation workers. Under conditions of high occupational stress, professional services cannot achieve the expected service effect, which greatly affects employees’ sense of work accomplishment and professional identity. The exhaustion caused by occupational emotion will lead to burnout and erode the quality of life of community rehabilitation workers, resulting in turnover intention.

### Altruism moderates and “reduces pressure” by intervening in occupational mental health

This study is not intended to deny the importance of altruistic values in the helping profession; in contrast, it points out that altruism has a nonnegligible moderating role in the complex psychological mechanism by which occupational stress affects turnover intention, and the findings clarify the vague understanding of the role of professional values in vocational development in previous studies [72–74]. For community rehabilitation workers, although altruism cannot directly affect work pressure and turnover intention, it can positively moderate and decrease turnover intention by reducing the effects of work pressure on burnout and quality of life. By employing self-regulation strategies based on altruistic values, community rehabilitation workers can more effectively cope with the burnout caused by occupational stress, mitigate the impact of occupational stress on their quality of life, and reduce their turnover intention. This paper further confirms the effectiveness of self-regulation theory in explaining and predicting individual behaviour, particularly highlighting the potential indirect moderating role of altruism in the relationship between occupational stress and turnover intention, which is one of the key innovative findings of this study.

Improving the altruistic value of community rehabilitation workers still plays a very important role in stabilizing the team. Nevertheless, reducing occupational stress alone may not achieve the effect of altruistic value guidance controlling turnover intention, and it can only play a role in employees’ burnout and quality of life. Rational choice theory points out that workers engaged in professional helping are also rational people who need to obtain internal and external remuneration equal to the value of their emotional labour so that they will not ultimately leave their jobs in career vacillation. Workload reduction and vocational training alone cannot restrain the turnover intention of community rehabilitation workers. The most important approach is to focus on existing burnout and the quality of life of workers, understand the personalized psychological needs of community rehabilitation workers and provide continuous and diversified occupational mental health support.

### Limitations and conclusion

While our study provides valuable insights into altruism, several limitations should be noted. First, the cross-sectional nature of our study limits our ability to establish causal relationships between variables. Unlike longitudinal studies, which track changes over time, our data represent a snapshot in time, thus preventing us from drawing conclusions about temporal trends or causal mechanisms. Second, this study collected data from only 82 community rehabilitation workers in one city, so the sample size is also limited, which may affect the generalizability of our results. In addition, this study is limited to community rehabilitation workers and does not cover other types of helping workers, such as workers providing assistance to the aged, the young and judicial correction. Notably, whether the research findings can be universally applied requires cross-occupational comparative research in the future.

In summary, from the perspective of the occupational values of altruism, this study explores the moderating role of altruism hidden in the complex structural path between occupational stress, burnout, quality of life and turnover intention. The findings have positive practical value for determining how to correctly and effectively stabilize grassroots rehabilitation teams and improve the welfare of disabled people in communities by advocating altruistic values.

### Electronic supplementary material

Below is the link to the electronic supplementary material.

Supplementary Material 1

## Data Availability

The original data used to support the findings of this study have been deposited in the FIGSHARE repository (DOI: 10.6084/m9.figshare.25709262.v2).
